# Antifibrotics in the Management of Rheumatoid Arthritis-Associated Interstitial Lung Disease: Prospective Real-World Experience From an Interstitial Lung Disease Clinic in India

**DOI:** 10.7759/cureus.63518

**Published:** 2024-06-30

**Authors:** Ajoy K Behera, Vikas Kumar, Pratibha Sharma, Ranganath Ganga, Jhasaketan Meher, Saroj Pati, Kulshreshth Sinha

**Affiliations:** 1 Pulmonary Medicine, All India Institute of Medical Sciences, Raipur, Raipur, IND; 2 Microbiology, Balaji Institute of Medical Sciences, Raipur, IND; 3 General Medicine, All India Institute of Medical Sciences, Raipur, Raipur, IND; 4 Radiodiagnosis, All India Institute of Medical Sciences, Raipur, Raipur, IND

**Keywords:** connective tissue disease-associated interstitial lung disease, rheumatoid arthritis, pirfenidone, lung function test, nintedanib

## Abstract

Background & Objectives: Interstitial lung disease (ILD) in rheumatoid arthritis (RA) is a serious complication with varied prevalence ranging from 4% to as high as 68%, with varied presentation. Immunosuppressants and antifibrotics are used in the management of RA ILD. The clinicodemographic profile and presentation in our country need to be further explored. We assessed the efficacy and safety profile of antifibrotic drugs in combination with immunosuppressants among RA ILD patients.

Methods: A prospective observational study was conducted in the Interstitial Lung Disease (ILD) Clinic in the Department of Pulmonary Medicine, All India Institute of Medical Sciences Raipur, India, between January 2022 to January 2023. RA patients with dyspnea and chronic cough were referred to us for evaluation of ILD. Patients underwent clinical examination, complete lung function study including spirometry, single breath diffusion capacity for carbon monoxide (DLCO), six-minute walk test, and high-resolution computed tomography of the thorax. Quality of life was assessed using the King's Brief Interstitial Lung Disease (KBILD) questionnaire.

Results: Two hundred eighteen RA patients were evaluated and out of these, 43 (20.8%) had features of ILD on high-resolution computed tomogram (HRCT) thorax. Twenty-six (2.18%) met the inclusion criteria for starting antifibrotics. The mean ± SD. age of the patients was 52.96 ± 14.04 and the majority (77%) were females. Fourteen (53.38%) patients had usual interstitial pneumonia (UIP)/probable UIP pattern and 12 (46.22%) had nonspecific interstitial pneumonia (NSIP) patterns on HRCT. Out of 26 patients, 24 (92.3%) were started on antifibrotics. Fourteen (53.8%) patients were on nintedanib and 10 (38.4%) were on pirfenidone. The mean ± SD forced vital capacity (FVC)% predictedwas 62.5 ± 20.04. The mean ± SD. The DLCO percentage predicted was 54.4 ± 22.8. Twenty-two (84.6%) patients did not experience any side effects. The mean ± SD. KBILD score was 59.9 ± 11.17 and was similar in both sexes.

Conclusion: In our study, the prevalence of RA ILD was nearly 20.8% and more common in females. Twenty-four (2%) patients were included for antifibrotic treatment. There was an improvement in lung function at the end of six months, but the change was not significant. All patients tolerated antifibrotics well without any serious adverse events.

## Introduction

Interstitial lung disease (ILD) is a debilitating extra-articular manifestation of rheumatoid arthritis (RA). The prevalence of ILD among RA varied from 4% to 60% [1-4]. The risk factors for the development of ILD include male sex, older age, tobacco use, higher RA disease activity, extra-articular disease features (e.g., subcutaneous nodules), and seropositivity for RA autoantibodies like RA factor (RF) and anti-citrullinated protein antibodies (anti-CCP) [5]. The median survival of RA ILD varied from 3 to 10 years [4]. The treatment of RA ILD relies upon immunosuppressants [6].

The role of antifibrotics is well-established in progressive fibrosing ILD (PF ILD) [7]. In patients with RA is one of the causes of PF ILD, and there is an increased risk of mortality with the usual interstitial pneumonia (UIP) pattern on high-resolution computed tomogram (HRCT) and quantitative fibrosis score of more than 12% [8,9]. Antifibrotics have proven to slow the disease progression. The combined usage of immunosuppressants and antifibrotics was well tolerated by the patients [10,11]. Pirfenidone is an orally active synthetic molecule with antifibrotic, anti-inflammatory, and antioxidant properties. It acts by inhibiting transforming beta-induced differentiation of human lung fibroblasts and modulating procollagen synthesis. A pooled analysis of the results of CAPACITY 1, CAPACITY 2, and ASCEND trials showed that there was a significant decline in mortality rates and forced vital capacity (FVC) decline in the pirfenidone group. Additional benefits for progression-free survival, dyspnea, and a 6-minute walk distance were reported. TRAIL1 and studies by Solomon et al. showed that pirfenidone is useful in RA ILD to slow the rate of decline in FVC [12]. Nintedanib is a potential inhibitor of tyrosine kinase inhibitors including fibroblast growth factor, platelet-derived growth factor, and vascular endothelial growth factor. Nintedanib was first approved for systemic sclerosis (SSc) in Japan and the USA in 2019 in SSc ILD. Thirteen percent of the study population in the INBUILD trial were of RA ILD, and nintedanib slowed the rate of decline of FVC among PF ILD [10]. In the TRAIL1 study compared with the placebo group, patients in the pirfenidone group had a slower rate of decline in lung function, measured by the estimated annual change in absolute FVC (-66 vs -146; p=0·0082) and FVC% (-1·02 vs -3·21; p=0·0028). A study showed that pirfenidone can be used safely among RA ILD patients to slow the fall in FVC [12]. Mean predicted percentage of FVC in the INBUILD trial was 68.7±16 and the adjusted rate of decline in FVC was −79.0 ml per year in the nintedanib group and −154.2 ml per year in the placebo group [10]. Many of our patients present with much poorer lung function and no baseline HRCT to compare, with only a history of clinical worsening. There is limited evidence on timing the usage of antifibrotics among RA ILD patients in our country.

## Materials and methods

Aim

To study the impact of antifibrotic drugs on lung function over a period of 6 months along with standard background therapy in RA ILD patients.

Objectives

1) Study of the effect of antifibrotics on lung function in patients with RA ILD over 6 months’ duration, 2) Safety profile and tolerability of antifibrotics in patients with RA ILD

Ethics

A prospective observational study was conducted in the ILD Clinic in the Department of Pulmonary Medicine, All India Institute of Medical Sciences Raipur, India, between January 2022 and January 2023, after the Institutional Ethical Committee approval (Approval number: AIIMSRPR/IEC/2020/551). RA patients with dyspnea and chronic cough were referred to the ILD clinic for further evaluation.

Inclusion criteria

1) Confirmed case of RA, referred by the rheumatology division of Internal Medicine department of the institute for ILD evaluation, 2) patients with evidence of ILD on HRCT thorax performed at the time of assessment or within the last 3 months at the time of enrollment into the study. HRCT thorax showing at least 10% of total lung volume involvement as assessed by an eyeballing technique by a practicing radiologist.

Exclusion criteria

1) Patients with a history of bronchial asthma, chronic obstructive pulmonary disease, bronchiectasis, and post-tubercular pulmonary sequelae, 2) patients already on antifibrotics for RA ILD for more than 2 weeks at the initial time of enrollment, 3) contraindications or unable to perform lung function tests like hemoptysis, pneumothorax, or recent abdominal surgery.

Methodology

Patients underwent detailed clinical examination and data regarding the clinico-demographic, serologic profile, and history of treatment with immunosuppressants were collected. These patients underwent complete lung function tests (spirometry, whole-body plethysmography, diffusion capacity for carbon monoxide (DLCO), and a six-minute walk test) [13,14]. The King's Brief Interstitial Lung Disease (KBILD) questionnaire was used to assess their quality of life [15].

Lung function tests were carried out in Power Cube Body+ (with Diffusion+) (GANSHORN Medizin Electronic, Germany) as per the American Thoracic Society (ATS)-European Respiratory Society (ERS) recommendations [13,14], lung volumes such as residual volume (RV), functional residual capacity (FRC), total lung capacity (TLC) and DLCO determined by whole-body plethysmography [16]. A six-minute walk test was performed as per standard ATS recommendations [16]. Patients were started on antifibrotics (either pirfenidone or nintedanib) after the initial assessment and the choice of antifibrotics was made as per patient preference. Patients were followed up monthly, repeat KBILD scores, and lung functions were assessed after 6 months.

Statistical analysis

The data was analyzed using the IBM SPSS version 20.0 (IBM Corp, Armonk, USA). The quantitative variables were expressed in mean and standard deviation. Categorical data was expressed in the percentages. Paired t-test was used to calculate the differences between the groups. P-value <0.05 was taken as a cut-off for statistically significant differences.

## Results

Two hundred eighteen RA patients were referred to the ILD clinic, and 43 (20.8%) had features of ILD on the HRCT thorax. Twenty-six (2.18%) patients were included for antifibrotic treatment. The mean ± SD. age of the enrolled patients was 52.96 ± 14.04 years. There were 20 (76.92%) females and six (23.08%) males. Twenty (76.9%) patients belonged to lower socio-economic strata. The mean ± SD. BMI was 25.2± 5.59 kg/m^2^. The mean ± SD. baseline FVC % predicted was 62.5 ± 20.04. The mean ± SD. baseline forced expiratory volume in 1 second (FEV1)% predicted was 61.2 ± 22.12. The mean ± SD. baseline DLCO% predicted was 70.1 ± 15.2. The mean ± SD. baseline KBILD score was 59.9 ± 11.17. Table 1 shows the clinic demographic features of the patients in the study.

**Table 1:** Clinical, radiological, and serological profile of patients RA: rheumatoid arthritis; anti-CCP: anti-citrullinated protein; ANA: antinuclear antibody; SSc: systemic sclerosis; HRCT: high-resolution computed tomogram; MMF: mycophenolate mofetil

Characteristics	N (%)
RA factor positive	26 (100%)
Anti-CCP positive	26 (100%)
ANA positivity	7 (26.9%)
RA – SSc overlap	2 (7.7%)
HRCT pattern	
Usual interstitial pneumonia	14 (53.8%)
Nonspecific interstitial pneumonia	12 (46.1%)
Background immunosuppressants	
Steroids	13 (50%)
Methotrexate	9 (34.6%)
MMF	2 (7.7%)
Rituximab	2 (7.7%)
Patients initiated on antifibrotics (n=24)	
Pirfenidone	10 (41.7%)
Nintedanib	14 (58.3%)

Serologic profile

RA factor (RF) was positive in all patients, mean RF was 119.4 (95% CI: 57.8-181), mean RF in males and females was 93.5 and 205.8, respectively. Mean anti-CCP antibody was 218.5 (95% CI: 94.3-342.8), mean anti-CCP in males and females was 172.5 and 371.9, respectively. Antinuclear antibody was positive in 26% of patients and anti-cytoplasmic antibody was in all the patients.

Radiologic and lung function profile

53.38% (14) had UIP/probable UIP pattern and 46.22% (12) had NSIP pattern on HRCT. Mean FEV1 was 1.48 l (95% CI: 1.30-1.66), mean FVC was 1.80 l (95% CI: 1.58-2.02), mean DLCO percentage of predicted was 48.58 (95% CI: 38.79-58.36). Table 1 shows the HRCT thorax profile of all the patients included in the study.

Antifibrotics safety and tolerance

Antifibrotics safety and tolerance 53.8% (14), 38.5% (10), and 7.7% (2) were on nintedanib, pirfenidone, and no antifibrotic treatment, respectively. Both the drugs were started at low doses and achieved full therapeutic doses of 150 mg/day and 1200 mg/day of nintedanib and pirfenidone, respectively, over four to six weeks’ duration. Beyond 1200 mg/day of pirfenidone patients were experiencing excessive gastrointestinal side effects. Gastrointestinal side effects were commonly observed in both groups and were managed with symptomatic medication. Two patients on pirfenidone experienced photosensitivity. Two patients stopped pirfenidone and did not want to be restarted due to intolerance. Table 2 shows the side effect profile of patients during the study.

**Table 2:** Adverse effect profile of antifibrotics among RA ILD patients RA ILD: rheumatoid arthritis interstitial lung disease

Adverse Effect	Nintedanib (n=14)%	Pirfenidone (n=10)
Nausea and vomiting	3 (21%)	3 (30%)
Loose stools	3 (21%)	1 (10%)
Loss of appetite	2 (14%)	2 (20%)
Liver dysfunction	2 (14%)	3 (30%)
Skin discoloration	0 (0%)	2 (20%)
Drug stopped due to intolerance	0 (0%)	2 (20%)

Lung function profile

All patients had mild restriction and mild diffusion defect at enrollment. On follow-up, there was no deterioration in lung function, rather there was a slight improvement in the lung function was noted, which was not statistically significant. There was no significant change in the quality of life at the end of the study period. Table 3 shows the comparison of changes in lung function at the end of the study period of 6 months.

**Table 3:** Comparisons of lung function at baseline and at 6 months of antifibrotic treatment *Data expressed in Mean ± SD. P< 0.05 is considered significant. FVC: forced vital capacity; TLC: total lung capacity; DLCO: diffusion capacity for carbon monoxide; 6mwD: 6-minute walk distance; KBILD: the King's Brief Interstitial Lung Disease

PFT Parameter*	At Baseline	At 6 Months	P-value
FVC (% predicted)	62.5 ± 20.04	63.2 ± 18.2	0.3
TLC (% predicted)	54.4 ± 22.8	56.1 ± 21.3	0.2
DLCO (% predicted)	70.1 ± 15.2	72.1 ± 12.4	0.15
6mwD (meters)	263 ± 40.1	280 ± 22.2	0.07
KBILD	59.9 ± 11.17	61.2 ± 13.4	0.3

## Discussion

RA ILD is one of the extra-articular pulmonary manifestations of RA. The presence of ILD among RA patients affects the prognosis of the patients [2]. The treatment of RA ILD patients primarily relies upon immunosuppressants [5]. At present, antifibrotics are used as a standard of care in idiopathic pulmonary fibrosis and progressive fibrosis subsets of ILD [17]. Our study studied the efficacy and safety of antifibrotics among RA ILD patients. We found that RA ILD was common among the elderly population, majority of patients were females similar to a study by Chang et al. where 67% of their study were females [18]. Bongartz et al. also found that the elderly population was mostly affected by male predilection [2]. Females had higher titers of RA factor and anti-CCP when compared with their male counterparts.

Prevalence of ILD in RA is quite variable, in our study 20.8% of RA patients had ILD. Sparks et al. found 5% of RA patients developed ILD. They included 509 787 RA patients,76.2% were females [1]. Gabbay et al. and Bongartz et al. found a prevalence of 58% and 8%, respectively [19,2]. In India, Gauri et al. found the prevalence of ILD as 60% [20].

HRCT chest was used to screen these patients for ILD. HRCT chest has sensitivity of 20-60% for detecting ILD [21]. Common ILD patterns in RA are UIP, NSIP, organizing pneumonia, diffuse alveolar damage (DAD), lymphocytic interstitial pneumonia (LIP), and follicular bronchiolitis [22]. In our study, the most common pattern radiologically was the UIP pattern (53%) and other studies also report UIP pattern was predominant followed by the NSIP pattern [1,5,18].

In our study, 26 patients of RA ILD were with lung involvement more than 10% of total lung volume on HRCT thorax. Twenty-four patients received antifibrotics (10 patients received pirfenidone and 14 received nintedanib). On a follow-up of 6 months, there was minimal improvement in lung function parameters, but it was not statistically significant. There was no decline in FVC and DLCO over 6 months of follow-up. Studies have shown that the use of antifibrotics stabilizes lung functions, and the incidence of major adverse events is less [11]. INBUILD Trail demonstrated safety and efficacy among the PF ILD patients. In this study, RA ILD patients also had representation. The rate of FVC decline was less as compared to placebo with a favorable safety profile [10]. Another study demonstrated the efficacy and safety of pirfenidone among RA ILD patients. Compared to the placebo, a slower rate of decline of FVC was observed and no serious adverse events were observed [12]. Among idiopathic pulmonary fibrosis (IPF) patients, pirfenidone improved the quality of life [23]. However, there is no evidence to demonstrate the effect of antifibrotics on the quality of life among RA ILD patients. In our study, there was no significant change in KBILD scores over 6 months. Most of our patients experienced gastrointestinal side effects which were managed by slow titrations of drug dosage, only two (8.3%) patients stopped the drug due to adverse effects. The rate of adverse events was low as compared to previous studies [7,11,12].

Limitations of the study

Our study had a few limitations. This study was a single-center study and referral bias could not be eliminated. The study participants were already on immunosuppressant therapy before enrollment, therefore the isolated effects of antifibrotics could not be studied. Also, the long-term follow-up of these patients is required.

## Conclusions

The antifibrotics are currently recommended for use among the PF ILD subsets of RA. No guidelines had advocated the use of antifibrotics at baseline among the RA ILD patients. Certain issues need to be addressed. First, no definite markers are available which may predict the progression of the disease. Second, the UIP pattern and increased lung fibrosis score carry a poor prognosis. Therefore, initiation of antifibrotics along with immunosuppressants may halt the disease progression. Hence, our study concluded that early initiation of antifibrotics among RA ILD patients with either UIP pattern or increased fibrosis score may be beneficial in stabilizing lung functions without an increase in adverse events.
